# Editorial

**Published:** 1860-11

**Authors:** 


### EDITORIAL.

### MEDICAL SCHOOLS IN CHICAGO.

*Lind University.*—The Medical Department of Lind University commenced its Second Annual Course of Lectures on Monday, October 7th. Prof. W. H. Byford delivered the general introductory Lecture, to a very full and intelligent audience, composed of Students, members of the Profession, and citizens. The incentives to high attainments in the science and practice of Medicine, constituted the theme of the Lecturer; and these were developed and urged upon the attention of the Students, in a manner both pleasing and profitable. The full Course has now been progressing four weeks, with perfect regularity and satisfaction in all its departments, and the number in attendance shows a very gratifying increase over the class of last year. We have learned that this increase would have been much greater, had it not been for an impression circulated industriously, to the effect that Students attending their first course in the Junior department of this University, would not receive credit for a full course, if they should wish to attend a second and graduate in any of the schools in Philadelphia or New-York. If there ever was any foundation for this story, it is entirely removed by the more perfect arrangements of the present term. For while the Faculty strictly adhere to the original rule of holding all junior students responsible for close attention to, and a thorough examination on, the important branches specially included in the junior department, they have so arranged the lecture hours that they are also privileged to attend all the Lectures on the practical branches. They will thus become entitled to, and will receive, a certificate of attendance on a full course of *five* months, including all the branches usually taught in the best Medical Colleges in this country.

---

*Rush Medical College.*—We are informed that the regular Annual Course of Lectures in this institution will commence on Wednesday evening, Nov. 7th. The number of Students attending the preliminary Lectures up to the present time, indicate a class for the full term of about the same number as in former years.

---

*Hahnemann Medical College.*—Under this title, the practitioners of Homœopathy in this city, during the past summer, organized a Faculty, fitted up Lecture rooms, got the usual puffs in the newspapers, issued numerous circulars, and finally when the eventful day was near at hand on which they had fixed for opening the institution, they advertised a whole week of Introductory Lectures. The week came and passed away, and with it *three Students*, one of whom had been previously appointed "Demonstrator of Anatomy." But as all three of the students wanted their tickets on credit, we believe the Faculty has never progressed in its course beyond the introductories. In other words, the "Hahnemann Medical College, of Chicago," stopped just after it started. We hope our friends of the Hahnemannic order will not be discouraged. We would suggest to them an appeal to the Ladies for another *benefit.*

### CHICAGO ACADEMY OF MEDICAL SCIENCES.

REGULAR MEETING, NOV., 2d, 1860.

DR. HAMILL, President, in the Chair.

After the usual preliminaries, Dr. M. O. Haydock, who had been appointed to deliver the anniversary address, read a paper on Leucosythema. Some discussion was elicited by this paper, from which it would appear the disease in question is of very rare occurrence in this city. We hope to give the paper of Dr. Haydock entire in our next issue.

*Hooping-Cough* constituted the special topic for discussion at this meeting; and elicited remarks from Drs. Bevan, Bloodgood, Haydock, Schlœtzer, Hamill, and Davis.

Dr. Bloodgood stated that he had used the Belladonna in many cases of this disease; in some with prompt benefit in lessening the severity of the cough, and apparently shortening the duration of the disease, while in others it seemed to exert no influence. He had found the extract more efficacious than the tincture. Dr. Heydock had met with the same variable results from the use of Belladonna, but had generally found benefit from the use of cochineal with the alkalies. Dr. Schœltzer, stated that he had usually seen Hooping-Cough promptly and permanently relieved by feeding the patients on meet and rich animal broths with plenty of wine. He recommended to a child two years old half a pint of wine per day. Dr. Davis inquired whether the difference in the effect of Belladonna and other narcotics on different cases of this diseae, was not owing to diverse pathological conditions of the air passages? For while the disease was doubtless essentially spasmodic and dependant on a peculiar irritation established in certain nerve structures, there often existed coincidently, especially during the first two weeks, a low grade of irritation in the bronchial mucous membrane. When such was the case, he had not found Belladonna or any other narcotic to act beneficially unless combined with an expectorant. The discussion was maintained with interest until the hour of adjournment.

### SCOTT COUNTY MEDICAL SOCIETY.

The Scott County Medical Society held its regular quarterly meeting at the City Hall, Davenport, on Tuesday, Oct. 30th, commencing at 10 o'clock A. M., a fair number of members being in attendance.

The President, Dr. Gamble, being absent, the Vice President, Dr. Witherwax, took the Chair.

Dr. Lyman Carpenter, of Blue Grass, stated through the Censors that it was his intention to remove from that State, and requested of the Society honorable dismissal. Action upon the matter was postponed till the afternoon session.

After the transaction of other business the Society adjourned till 2 o'clock P. M.

AFTERNOON SESSION.

Dr. Gamble, the President, appeared and called the Society to order.

The request of Dr. Carpenter, made at the morning session, was unanimously complied with, and the President and Secretary were directed to issue an appropriate card of dismissal.

Dr. Barrows then offered the following preamble and resolution, which were adopted unanimously:

*Whereas*, Dr. Lyman Carpenter, one of the founders as well as one of the most respected members of this Society, has requested and had granted an honorable dismissal, for the reason that he is about to remove from the State of Iowa; therefore,

*Resolved*, That this Society cannot but regard with pleasure the relations which have been uniformly maintained with our departing brother, and regret that it has become necessary for him to dissolve his connection with us; also, that Dr. Carpenter carry with him those assurances, upon our part, of continued interest and regard which a consistant medical career in our midst so eminently deserve.

Dr. Fountain presented a case of recovery from hip joint disease in the person of a child nine years of age, the same having been effected under the influence of a mode of treatment which Dr. F. detailed at length.

Dr. Parry read a paper describing a fatal case of poisoning occasioned by swallowing the seeds of the stramonium (thorn-apple or Jamestown weed). The reading of the paper elicited considerable discussion, and many members stated cases of a similar character coming under their observation, although not always with fatal results.

In connection with the subject, resolutions were offered by Dr. Parry, and adopted, requesting the City Council of Davenport to declare the poisonous weed a nuisance, and to take measures for its extirpation.

Dr. Fountain offered the following preamble and resolution:

*Whereas*, The medical profession are everywhere cognizant of the fact that the crime of *criminal abortion* is fearfully prevalent, and increasing in all classes of society; and

*Whereas*, The progress of civialization and the spread of religion appear not to have had the effect of diminishing this species of iniquity; therefore be it

*Resolved*, 1. That the members of this Society will co-operate with the Ameriean Medical Association and other organizations of the kind in using every effort to disseminate a knowledge of the criminal nature of practices which are too often regarded as harmless, and frequently resorted to by many who would shudder at the thought of destroying the life of a human being.

2. *Resolved*, That the members of this Society unite in sentiment with the opinion of the best and most learned men of the profession in all parts of the world, that the fœtus is a living being from the earliest period of gestation, the willful destruction of which, except when required for the preservation of the life of the mother, is a crime as monstrous as infanticide, and its perpetrators should be regarded as felons by the laws of man, as they must be by every precept of morality.

3. *Resolved*, That every member of this Society who may be known to yield to the solicitations of any party for the purposes above indicated, shall forfeit his membership and be regarded as unworthy of fellowship by all honorable physicians.

4. *Resolved*, That it shall be considered the duty of every physician, when application for such purpose is made, not only to decline promptly, but to exert his personal influence to the utmost to prevent its accomplishment, by explaining its criminal character and removing as far as possible the erroneous opinions which are so generally prevalent regarding the life of the fœtus.

5. *Resolved*, That we denounce the common practice of newspaper proprietors in publishing advertisements which are calculated to encourage the practice of criminal abortion, as one prolific cause of a vast amount of crime and immorality, for which such newspaper editors and proprietors are thereby in a great degree responsible.

6. *Resolved*, That we likewise denounce the practice of many druggists in keeping for sale and dispensing such preparations as are kuown to be used for the purpose of producing abortion, which practice is no less reprehensible than to furnish poison when knowingly purchased with murderous intent, and by which all such druggists are *participis criminis* in the evil work of corrupting good morals, and willfully engaged in aiding and assisting in the perpetration of a crime which should be held in abhorrence by every member of a civilized and Christian community.

Which, having been read and commented upon, were unanimously adopted:

The application of Dr. Bowen, of Le Claire, for membership was laid over till the next meeting.

Dr. Baker was continued as essayist, after having rendered a satisfactory excuse for non-performance of duty at the present time.

Drs. Fountain, Keith and Adler, were appointed the committee on prevailing diseases.

A vote of thanks to the City Council, for the use of the council-chamber, was adopted.

The Society then adjourned.

